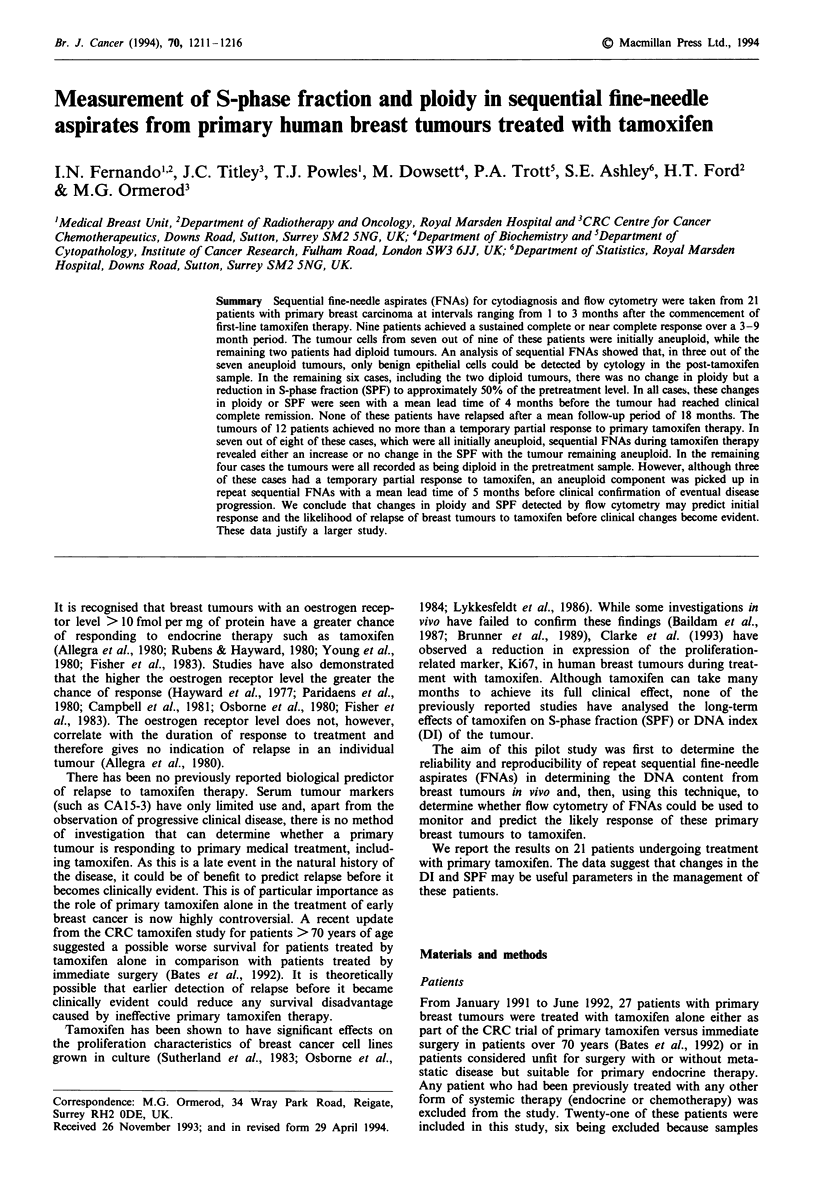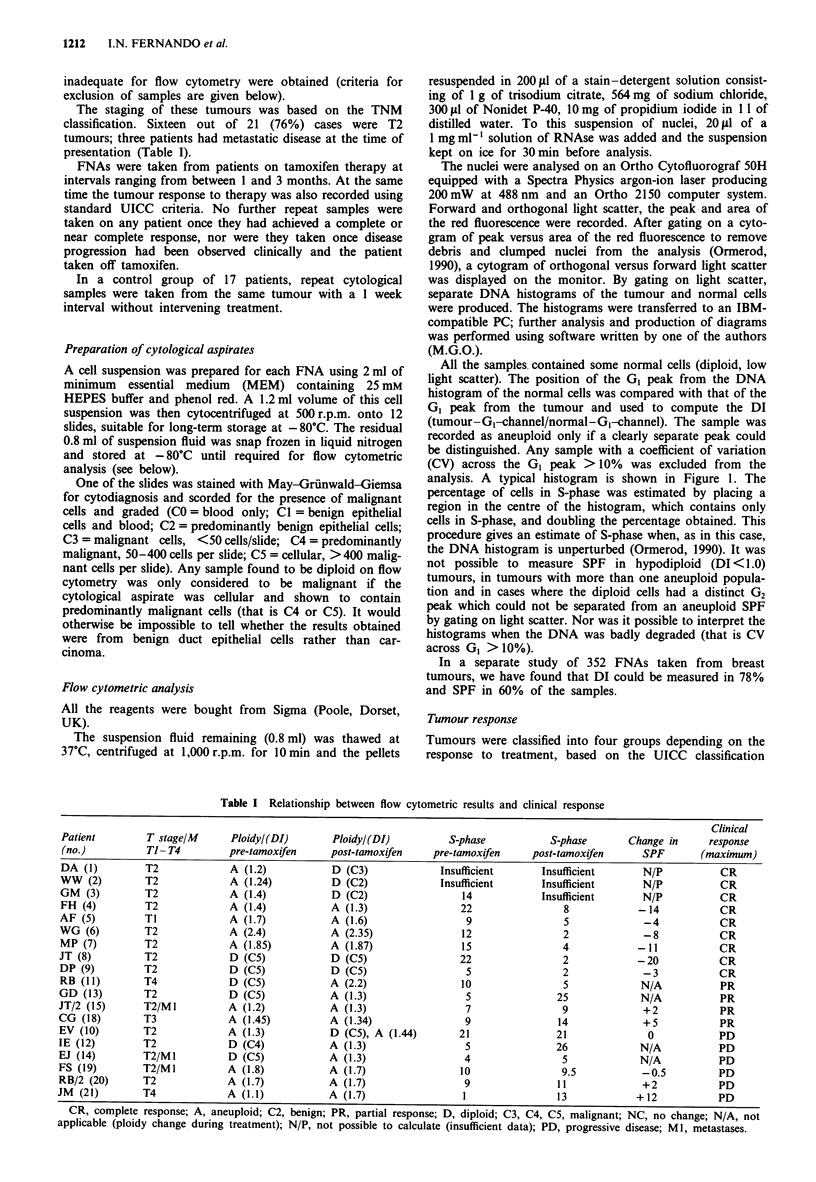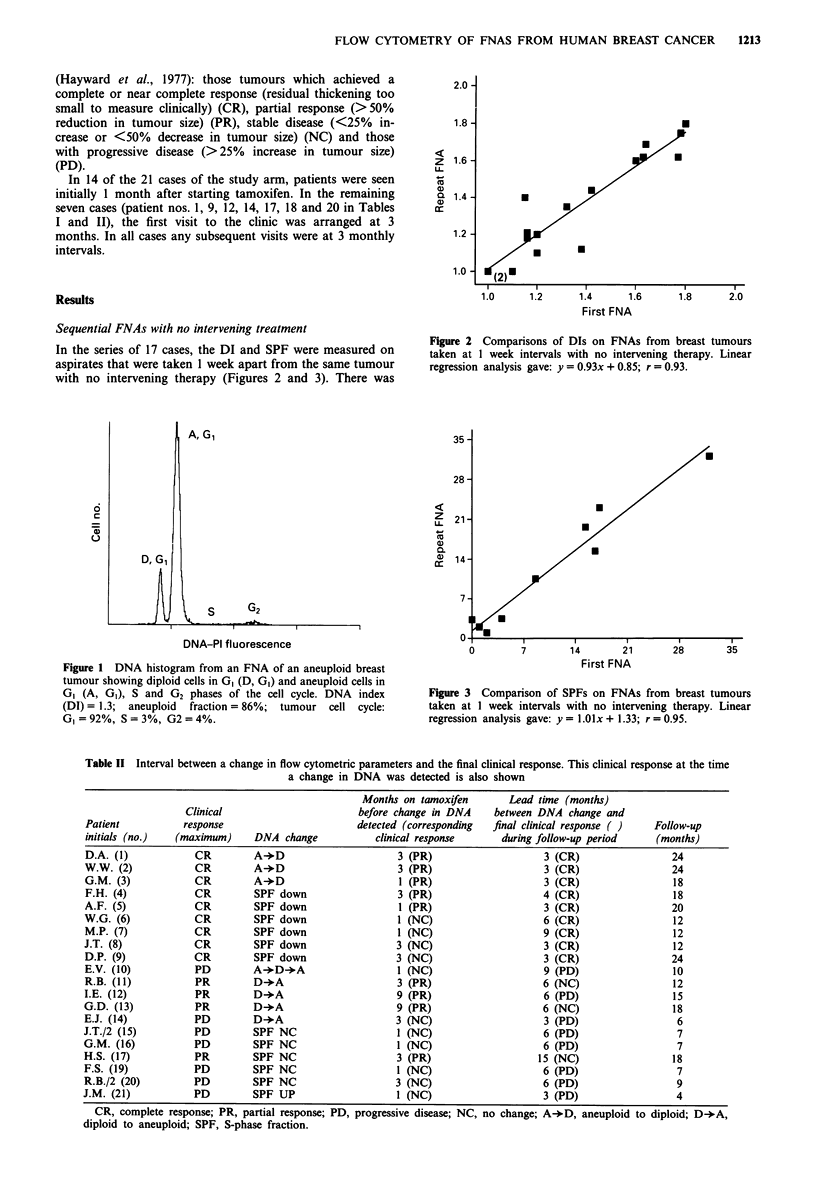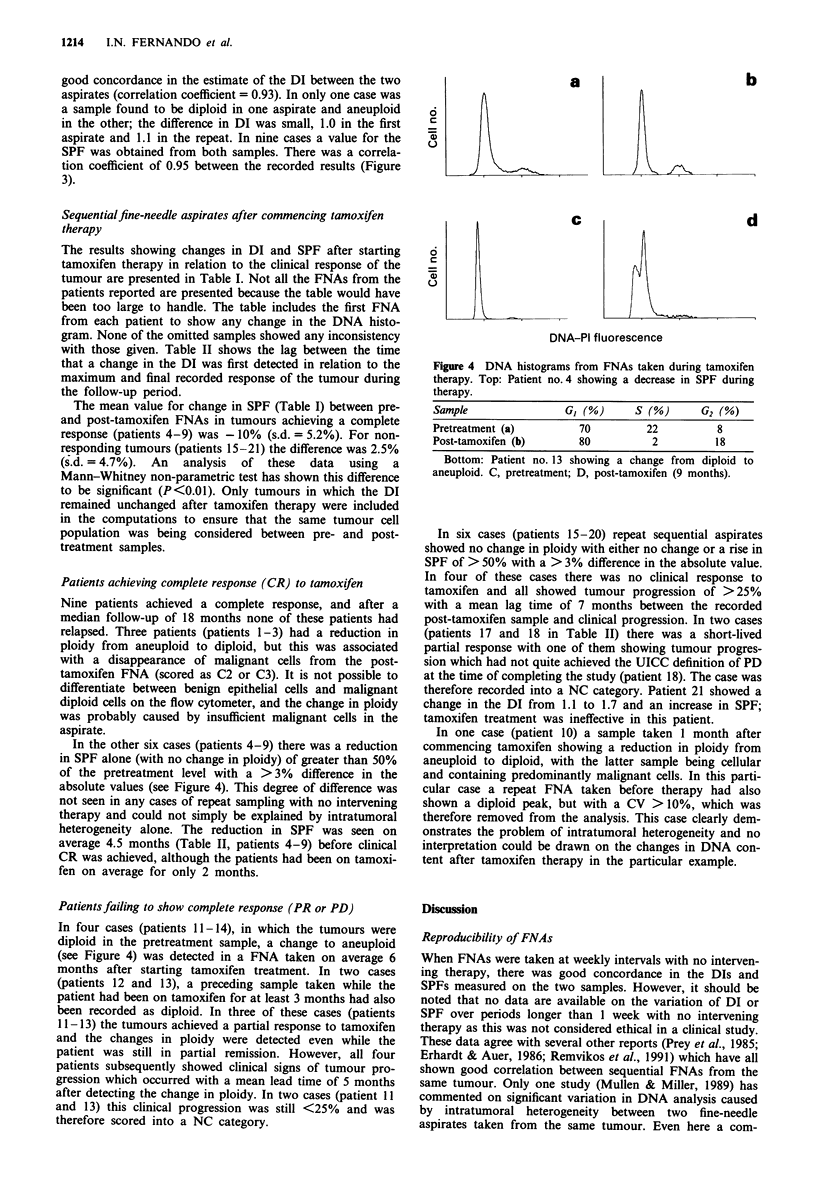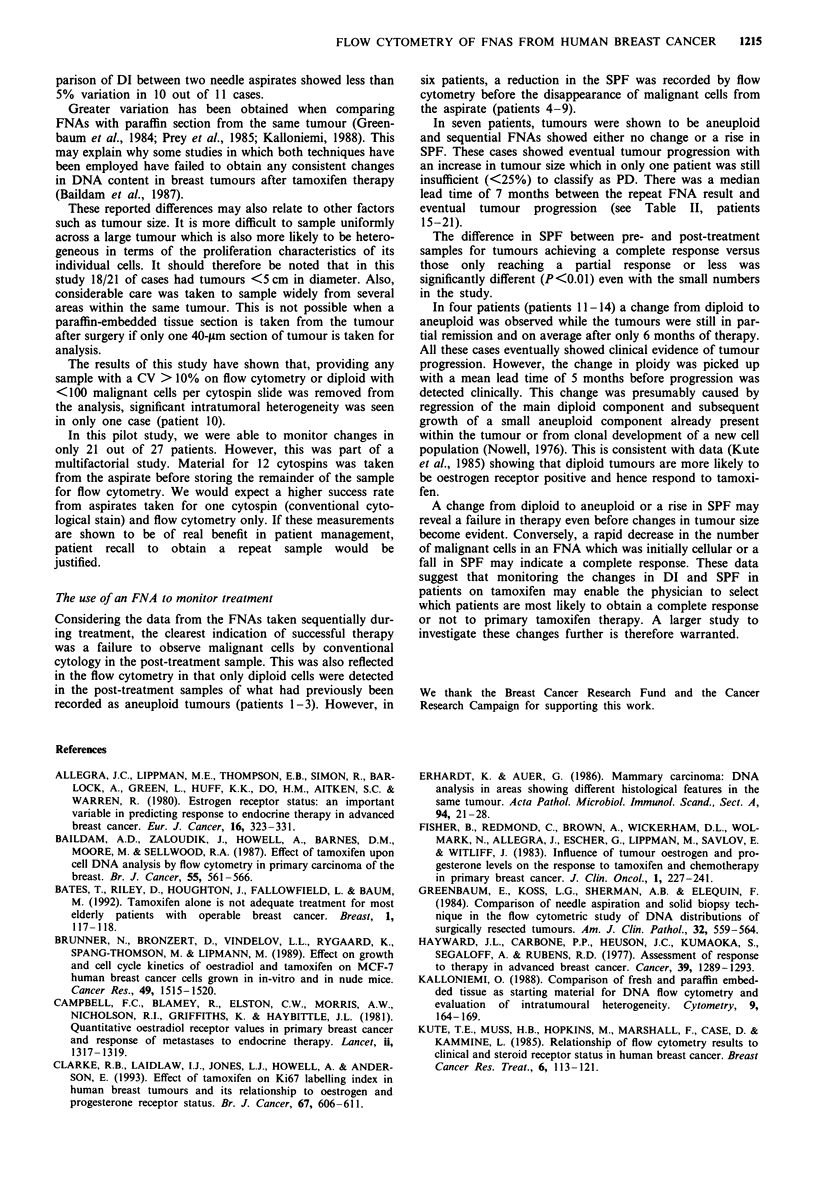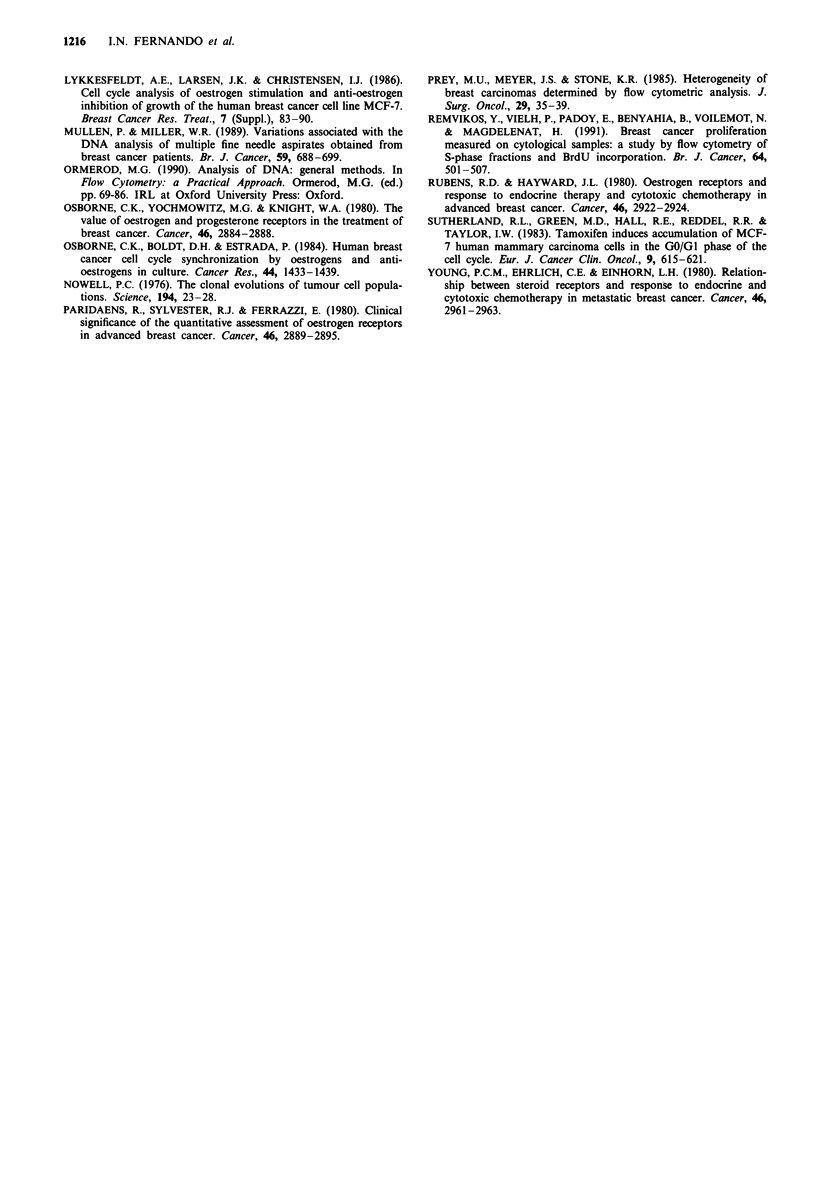# Measurement of S-phase fraction and ploidy in sequential fine-needle aspirates from primary human breast tumours treated with tamoxifen.

**DOI:** 10.1038/bjc.1994.475

**Published:** 1994-12

**Authors:** I. N. Fernando, J. C. Titley, T. J. Powles, M. Dowsett, P. A. Trott, S. E. Ashley, H. T. Ford, M. G. Ormerod

**Affiliations:** Department of Radiotherapy and Oncology, Royal Marsden Hospital, Surrey, UK.

## Abstract

Sequential fine-needle aspirates (FNAs) for cytodiagnosis and flow cytometry were taken from 21 patients with primary breast carcinoma at intervals ranging from 1 to 3 months after the commencement of first-line tamoxifen therapy. Nine patients achieved a sustained complete or near complete response over a 3-9 month period. The tumour cells from seven out of nine of these patients were initially aneuploid, while the remaining two patients had diploid tumours. An analysis of sequential FNAs showed that, in three out of the seven aneuploid tumours, only benign epithelial cells could be detected by cytology in the post-tamoxifen sample. In the remaining six cases, including the two diploid tumours, there was no change in ploidy but a reduction in S-phase fraction (SPF) to approximately 50% of the pretreatment level. In all cases, these changes in ploidy or SPF were seen with a mean lead time of 4 months before the tumour had reached clinical complete remission. None of these patients have relapsed after a mean follow-up period of 18 months. The tumours of 12 patients achieved no more than a temporary partial response to primary tamoxifen therapy. In seven out of eight of these cases, which were all initially aneuploid, sequential FNAs during tamoxifen therapy revealed either an increase or no change in the SPF with the tumour remaining aneuploid. In the remaining four cases the tumours were all recorded as being diploid in the pretreatment sample. However, although three of these cases had a temporary partial response to tamoxifen, an aneuploid component was picked up in repeat sequential FNAs with a mean lead time of 5 months before clinical confirmation of eventual disease progression. We conclude that changes in ploidy and SPF detected by flow cytometry may predict initial response and the likelihood of relapse of breast tumours to tamoxifen before clinical changes become evident. These data justify a larger study.


Br. J. Cancer (1994), 70, 1211–1216 © Macmillan Press Ltd., 1994

### Measurement of S-phase fraction and ploidy in sequential fine-needle aspirates from primary human breast tumours treated with tamoxifen

I.N. Fernando[1,2], J.C. Titley[3], T.J. Powles[1], M. Dowsett[4], P.A. Trott[5], S.E. Ashley[6], H.T. Ford[2] & M.G. Ormerod[3]

[1]*Medical Breast Unit,* [2]*Department of Radiotherapy and Oncology, Royal Marsden Hospital and* [3]*CRC Centre for Cancer Chemotherapeutics, Downs Road, Sutton, Surrey SM2 5NG, UK;* [4]*Department of Biochemistry and* [5]*Department of Cytopathology, Institute of Cancer Research, Fulham Road, London SW3 6JJ, UK;* [6]*Department of Statistics, Royal Marsden Hospital, Downs Road, Sutton, Surrey SM2 5NG, UK.*

**Summary** Sequential fine-needle aspirates (FNAs) for cytodiagnosis and flow cytometry were taken from 21 patients with primary breast carcinoma at intervals ranging from 1 to 3 months after the commencement of first-line tamoxifen therapy. Nine patients achieved a sustained complete or near complete response over a 3–9 month period. The tumour cells from seven out of nine of these patients were initially aneuploid, while the remaining two patients had diploid tumours. An analysis of sequential FNAs showed that, in three out of the seven aneuploid tumours, only benign epithelial cells could be detected by cytology in the post-tamoxifen sample. In the remaining six cases, including the two diploid tumours, there was no change in ploidy but a reduction in S-phase fraction (SPF) to approximately 50% of the pretreatment level. In all cases, these changes in ploidy or SPF were seen with a mean lead time of 4 months before the tumour had reached clinical complete remission. None of these patients have relapsed after a mean follow-up period of 18 months. The tumours of 12 patients achieved no more than a temporary partial response to primary tamoxifen therapy. In seven out of eight of these cases, which were all initially aneuploid, sequential FNAs during tamoxifen therapy revealed either an increase or no change in the SPF with the tumour remaining aneuploid. In the remaining four cases the tumours were all recorded as being diploid in the pretreatment sample. However, although three of these cases had a temporary partial response to tamoxifen, an aneuploid component was picked up in repeat sequential FNAs with a mean lead time of 5 months before clinical confirmation of eventual disease progression. We conclude that changes in ploidy and SPF detected by flow cytometry may predict initial response and the likelihood of relapse of breast tumours to tamoxifen before clinical changes become evident. These data justify a larger study.

It is recognised that breast tumours with an oestrogen receptor level > 10 fmol per mg of protein have a greater chance of responding to endocrine therapy such as tamoxifen (Allegra *et al.*, 1980; Rubens & Hayward, 1980; Young *et al.*, 1980; Fisher *et al.*, 1983). Studies have also demonstrated that the higher the oestrogen receptor level the greater the chance of response (Hayward *et al.*, 1977; Paridaens *et al.*, 1980; Campbell *et al.*, 1981; Osborne *et al.*, 1980; Fisher *et al.*, 1983). The oestrogen receptor level does not, however, correlate with the duration of response to treatment and therefore gives no indication of relapse in an individual tumour (Allegra *et al.*, 1980).

There has been no previously reported biological predictor of relapse to tamoxifen therapy. Serum tumour markers (such as CA15-3) have only limited use and, apart from the observation of progressive clinical disease, there is no method of investigation that can determine whether a primary tumour is responding to primary medical treatment, including tamoxifen. As this is a late event in the natural history of the disease, it could be of benefit to predict relapse before it becomes clinically evident. This is of particular importance as the role of primary tamoxifen alone in the treatment of early breast cancer is now highly controversial. A recent update from the CRC tamoxifen study for patients > 70 years of age suggested a possible worse survival for patients treated by tamoxifen alone in comparison with patients treated by immediate surgery (Bates *et al.*, 1992). It is theoretically possible that earlier detection of relapse before it became clinically evident could reduce any survival disadvantage caused by ineffective primary tamoxifen therapy.

Tamoxifen has been shown to have significant effects on the proliferation characteristics of breast cancer cell lines grown in culture (Sutherland *et al.*, 1983; Osborne *et al.*, 1984; Lykkesfeldt *et al.*, 1986). While some investigations *in vivo* have failed to confirm these findings (Baildam *et al.*, 1987; Brunner *et al.*, 1989), Clarke *et al.* (1993) have observed a reduction in expression of the proliferation-related marker, Ki67, in human breast tumours during treatment with tamoxifen. Although tamoxifen can take many months to achieve its full clinical effect, none of the previously reported studies have analysed the long-term effects of tamoxifen on S-phase fraction (SPF) or DNA index (DI) of the tumour.

The aim of this pilot study was first to determine the reliability and reproducibility of repeat sequential fine-needle aspirates (FNAs) in determining the DNA content from breast tumours *in vivo* and, then, using this technique, to determine whether flow cytometry of FNAs could be used to monitor and predict the likely response of these primary breast tumours to tamoxifen.

We report the results on 21 patients undergoing treatment with primary tamoxifen. The data suggest that changes in the DI and SPF may be useful parameters in the management of these patients.

### Materials and methods

### *Patients*

From January 1991 to June 1992, 27 patients with primary breast tumours were treated with tamoxifen alone either as part of the CRC trial of primary tamoxifen versus immediate surgery in patients over 70 years (Bates *et al.*, 1992) or in patients considered unfit for surgery with or without metastatic disease but suitable for primary endocrine therapy. Any patient who had been previously treated with any other form of systemic therapy (endocrine or chemotherapy) was excluded from the study. Twenty-one of these patients were included in this study, six being excluded because samples

Correspondence: M.G. Ormerod, 34 Wray Park Road, Reigate, Surrey RH2 0DE, UK.
Received 26 November 1993; and in revised form 29 April 1994.

inadequate for flow cytometry were obtained (criteria for exclusion of samples are given below).

The staging of these tumours was based on the TNM classification. Sixteen out of 21 (76%) cases were T2 tumours; three patients had metastatic disease at the time of presentation (Table I).

FNAs were taken from patients on tamoxifen therapy at intervals ranging from between 1 and 3 months. At the same time the tumour response to therapy was also recorded using standard UICC criteria. No further repeat samples were taken on any patient once they had achieved a complete or near complete response, nor were they taken once disease progression had been observed clinically and the patient taken off tamoxifen.

In a control group of 17 patients, repeat cytological samples were taken from the same tumour with a 1 week interval without intervening treatment.

*Preparation of cytological aspirates*

A cell suspension was prepared for each FNA using 2 ml of minimum essential medium (MEM) containing 25 mM HEPES buffer and phenol red. A 1.2 ml volume of this cell suspension was then cytocentrifuged at 500 r.p.m. onto 12 slides, suitable for long-term storage at −80°C. The residual 0.8 ml of suspension fluid was snap frozen in liquid nitrogen and stored at −80°C until required for flow cytometric analysis (see below).

One of the slides was stained with May–Grünwald–Giemsa for cytodiagnosis and scorded for the presence of malignant cells and graded (C0 = blood only; C1 = benign epithelial cells and blood; C2 = predominantly benign epithelial cells; C3 = malignant cells, <50 cells/slide; C4 = predominantly malignant, 50–400 cells per slide; C5 = cellular, >400 malignant cells per slide). Any sample found to be diploid on flow cytometry was only considered to be malignant if the cytological aspirate was cellular and shown to contain predominantly malignant cells (that is C4 or C5). It would otherwise be impossible to tell whether the results obtained were from benign duct epithelial cells rather than carcinoma.

*Flow cytometric analysis*

All the reagents were bought from Sigma (Poole, Dorset, UK).

The suspension fluid remaining (0.8 ml) was thawed at 37°C, centrifuged at 1,000 r.p.m. for 10 min and the pellets resuspended in 200 µl of a stain–detergent solution consisting of 1 g of trisodium citrate, 564 mg of sodium chloride, 300 µl of Nonidet P-40, 10 mg of propidium iodide in 1 l of distilled water. To this suspension of nuclei, 20 µl of a 1 mg ml$^{-1}$ solution of RNAse was added and the suspension kept on ice for 30 min before analysis.

The nuclei were analysed on an Ortho Cytofluorograf 50H equipped with a Spectra Physics argon-ion laser producing 200 mW at 488 nm and an Ortho 2150 computer system. Forward and orthogonal light scatter, the peak and area of the red fluorescence were recorded. After gating on a cytogram of peak versus area of the red fluorescence to remove debris and clumped nuclei from the analysis (Ormerod, 1990), a cytogram of orthogonal versus forward light scatter was displayed on the monitor. By gating on light scatter, separate DNA histograms of the tumour and normal cells were produced. The histograms were transferred to an IBM-compatible PC; further analysis and production of diagrams was performed using software written by one of the authors (M.G.O.).

All the samples contained some normal cells (diploid, low light scatter). The position of the $G_1$ peak from the DNA histogram of the normal cells was compared with that of the $G_1$ peak from the tumour and used to compute the DI (tumour-$G_1$-channel/normal-$G_1$-channel). The sample was recorded as aneuploid only if a clearly separate peak could be distinguished. Any sample with a coefficient of variation (CV) across the $G_1$ peak >10% was excluded from the analysis. A typical histogram is shown in Figure 1. The percentage of cells in S-phase was estimated by placing a region in the centre of the histogram, which contains only cells in S-phase, and doubling the percentage obtained. This procedure gives an estimate of S-phase when, as in this case, the DNA histogram is unperturbed (Ormerod, 1990). It was not possible to measure SPF in hypodiploid (DI<1.0) tumours, in tumours with more than one aneuploid population and in cases where the diploid cells had a distinct $G_2$ peak which could not be separated from an aneuploid SPF by gating on light scatter. Nor was it possible to interpret the histograms when the DNA was badly degraded (that is CV across $G_1$ >10%).

In a separate study of 352 FNAs taken from breast tumours, we have found that DI could be measured in 78% and SPF in 60% of the samples.

*Tumour response*

Tumours were classified into four groups depending on the response to treatment, based on the UICC classification

**Table I** Relationship between flow cytometric results and clinical response

| *Patient (no.)* | *T stage/M T1–T4* | *Ploidy/(DI) pre-tamoxifen* | *Ploidy/(DI) post-tamoxifen* | *S-phase pre-tamoxifen* | *S-phase post-tamoxifen* | *Change in SPF* | *Clinical response (maximum)* |
|---|---|---|---|---|---|---|---|
| DA (1) | T2 | A (1.2) | D (C3) | Insufficient | Insufficient | N/P | CR |
| WW (2) | T2 | A (1.24) | D (C2) | Insufficient | Insufficient | N/P | CR |
| GM (3) | T2 | A (1.4) | D (C2) | 14 | Insufficient | N/P | CR |
| FH (4) | T2 | A (1.4) | A (1.3) | 22 | 8 | −14 | CR |
| AF (5) | T1 | A (1.7) | A (1.6) | 9 | 5 | −4 | CR |
| WG (6) | T2 | A (2.4) | A (2.35) | 12 | 2 | −8 | CR |
| MP (7) | T2 | A (1.85) | A (1.87) | 15 | 4 | −11 | CR |
| JT (8) | T2 | D (C5) | D (C5) | 22 | 2 | −20 | CR |
| DP (9) | T2 | D (C5) | D (C5) | 5 | 2 | −3 | CR |
| RB (11) | T4 | D (C5) | A (2.2) | 10 | 5 | N/A | PR |
| GD (13) | T2 | D (C5) | A (1.3) | 5 | 25 | N/A | PR |
| JT/2 (15) | T2/M1 | A (1.2) | A (1.3) | 7 | 9 | +2 | PR |
| CG (18) | T3 | A (1.45) | A (1.34) | 9 | 14 | +5 | PR |
| EV (10) | T2 | A (1.3) | D (C5), A (1.44) | 21 | 21 | 0 | PD |
| IE (12) | T2 | D (C4) | A (1.3) | 5 | 26 | N/A | PD |
| EJ (14) | T2/M1 | D (C5) | A (1.3) | 4 | 5 | N/A | PD |
| FS (19) | T2/M1 | A (1.8) | A (1.7) | 10 | 9.5 | −0.5 | PD |
| RB/2 (20) | T2 | A (1.7) | A (1.7) | 9 | 11 | +2 | PD |
| JM (21) | T4 | A (1.1) | A (1.7) | 1 | 13 | +12 | PD |

CR, complete response; A, aneuploid; C2, benign; PR, partial response; D, diploid; C3, C4, C5, malignant; NC, no change; N/A, not applicable (ploidy change during treatment); N/P, not possible to calculate (insufficient data); PD, progressive disease; M1, metastases.

(Hayward *et al.*, 1977): those tumours which achieved a complete or near complete response (residual thickening too small to measure clinically) (CR), partial response (>50% reduction in tumour size) (PR), stable disease (<25% increase or <50% decrease in tumour size) (NC) and those with progressive disease (>25% increase in tumour size) (PD).

In 14 of the 21 cases of the study arm, patients were seen initially 1 month after starting tamoxifen. In the remaining seven cases (patient nos. 1, 9, 12, 14, 17, 18 and 20 in Tables I and II), the first visit to the clinic was arranged at 3 months. In all cases any subsequent visits were at 3 monthly intervals.

### Results

### *Sequential FNAs with no intervening treatment*

In the series of 17 cases, the DI and SPF were measured on aspirates that were taken 1 week apart from the same tumour with no intervening therapy (Figures 2 and 3). There was

**Figure 2** Comparisons of DIs on FNAs from breast tumours taken at 1 week intervals with no intervening therapy. Linear regression analysis gave: $y = 0.93x + 0.85$; $r = 0.93$.

**Figure 1** DNA histogram from an FNA of an aneuploid breast tumour showing diploid cells in $G_1$ (D, $G_1$) and aneuploid cells in $G_1$ (A, $G_1$), S and $G_2$ phases of the cell cycle. DNA index (DI) = 1.3; aneuploid fraction = 86%; tumour cell cycle: $G_1$ = 92%, S = 3%, G2 = 4%.

**Figure 3** Comparison of SPFs on FNAs from breast tumours taken at 1 week intervals with no intervening therapy. Linear regression analysis gave: $y = 1.01x + 1.33$; $r = 0.95$.

**Table II** Interval between a change in flow cytometric parameters and the final clinical response. This clinical response at the time a change in DNA was detected is also shown

| *Patient initials (no.)* | *Clinical response (maximum)* | *DNA change* | *Months on tamoxifen before change in DNA detected (corresponding clinical response* | *Lead time (months) between DNA change and final clinical response ( ) during follow-up period* | *Follow-up (months)* |
|---|---|---|---|---|---|
| D.A. (1) | CR | A→D | 3 (PR) | 3 (CR) | 24 |
| W.W. (2) | CR | A→D | 3 (PR) | 3 (CR) | 24 |
| G.M. (3) | CR | A→D | 1 (PR) | 3 (CR) | 18 |
| F.H. (4) | CR | SPF down | 3 (PR) | 4 (CR) | 18 |
| A.F. (5) | CR | SPF down | 1 (PR) | 3 (CR) | 20 |
| W.G. (6) | CR | SPF down | 1 (NC) | 6 (CR) | 12 |
| M.P. (7) | CR | SPF down | 1 (NC) | 9 (CR) | 12 |
| J.T. (8) | CR | SPF down | 3 (NC) | 3 (CR) | 12 |
| D.P. (9) | CR | SPF down | 3 (NC) | 3 (CR) | 24 |
| E.V. (10) | PD | A→D→A | 1 (NC) | 9 (PD) | 10 |
| R.B. (11) | PR | D→A | 3 (PR) | 6 (NC) | 12 |
| I.E. (12) | PR | D→A | 9 (PR) | 6 (PD) | 15 |
| G.D. (13) | PR | D→A | 9 (PR) | 6 (NC) | 18 |
| E.J. (14) | PD | D→A | 3 (NC) | 3 (PD) | 6 |
| J.T./2 (15) | PD | SPF NC | 1 (NC) | 6 (PD) | 7 |
| G.M. (16) | PD | SPF NC | 1 (NC) | 6 (PD) | 7 |
| H.S. (17) | PR | SPF NC | 3 (PR) | 15 (NC) | 18 |
| F.S. (19) | PD | SPF NC | 1 (NC) | 6 (PD) | 7 |
| R.B./2 (20) | PD | SPF NC | 3 (NC) | 6 (PD) | 9 |
| J.M. (21) | PD | SPF UP | 1 (NC) | 3 (PD) | 4 |

CR, complete response; PR, partial response; PD, progressive disease; NC, no change; A→D, aneuploid to diploid; D→A, diploid to aneuploid; SPF, S-phase fraction.

good concordance in the estimate of the DI between the two aspirates (correlation coefficient = 0.93). In only one case was a sample found to be diploid in one aspirate and aneuploid in the other; the difference in DI was small, 1.0 in the first aspirate and 1.1 in the repeat. In nine cases a value for the SPF was obtained from both samples. There was a correlation coefficient of 0.95 between the recorded results (Figure 3).

*Sequential fine-needle aspirates after commencing tamoxifen therapy*

The results showing changes in DI and SPF after starting tamoxifen therapy in relation to the clinical response of the tumour are presented in Table I. Not all the FNAs from the patients reported are presented because the table would have been too large to handle. The table includes the first FNA from each patient to show any change in the DNA histogram. None of the omitted samples showed any inconsistency with those given. Table II shows the lag between the time that a change in the DI was first detected in relation to the maximum and final recorded response of the tumour during the follow-up period.

The mean value for change in SPF (Table I) between pre- and post-tamoxifen FNAs in tumours achieving a complete response (patients 4–9) was −10% (s.d. = 5.2%). For non-responding tumours (patients 15–21) the difference was 2.5% (s.d. = 4.7%). An analysis of these data using a Mann–Whitney non-parametric test has shown this difference to be significant ($P<0.01$). Only tumours in which the DI remained unchanged after tamoxifen therapy were included in the computations to ensure that the same tumour cell population was being considered between pre- and post-treatment samples.

*Patients achieving complete response (CR) to tamoxifen*

Nine patients achieved a complete response, and after a median follow-up of 18 months none of these patients had relapsed. Three patients (patients 1–3) had a reduction in ploidy from aneuploid to diploid, but this was associated with a disappearance of malignant cells from the post-tamoxifen FNA (scored as C2 or C3). It is not possible to differentiate between benign epithelial cells and malignant diploid cells on the flow cytometer, and the change in ploidy was probably caused by insufficient malignant cells in the aspirate.

In the other six cases (patients 4–9) there was a reduction in SPF alone (with no change in ploidy) of greater than 50% of the pretreatment level with a >3% difference in the absolute values (see Figure 4). This degree of difference was not seen in any cases of repeat sampling with no intervening therapy and could not simply be explained by intratumoral heterogeneity alone. The reduction in SPF was seen on average 4.5 months (Table II, patients 4–9) before clinical CR was achieved, although the patients had been on tamoxifen on average for only 2 months.

*Patients failing to show complete response (PR or PD)*

In four cases (patients 11–14), in which the tumours were diploid in the pretreatment sample, a change to aneuploid (see Figure 4) was detected in a FNA taken on average 6 months after starting tamoxifen treatment. In two cases (patients 12 and 13), a preceding sample taken while the patient had been on tamoxifen for at least 3 months had also been recorded as diploid. In three of these cases (patients 11–13) the tumours achieved a partial response to tamoxifen and the changes in ploidy were detected even while the patient was still in partial remission. However, all four patients subsequently showed clinical signs of tumour progression which occurred with a mean lead time of 5 months after detecting the change in ploidy. In two cases (patient 11 and 13) this clinical progression was still <25% and was therefore scored into a NC category.

**Figure 4** DNA histograms from FNAs taken during tamoxifen therapy. Top: Patient no. 4 showing a decrease in SPF during therapy.

| *Sample* | $G_1$ (%) | *S (%)* | $G_2$ (%) |
|---|---|---|---|
| Pretreatment (**a**) | 70 | 22 | 8 |
| Post-tamoxifen (**b**) | 80 | 2 | 18 |

Bottom: Patient no. 13 showing a change from diploid to aneuploid. C, pretreatment; D, post-tamoxifen (9 months).

In six cases (patients 15–20) repeat sequential aspirates showed no change in ploidy with either no change or a rise in SPF of >50% with a >3% difference in the absolute value. In four of these cases there was no clinical response to tamoxifen and all showed tumour progression of >25% with a mean lag time of 7 months between the recorded post-tamoxifen sample and clinical progression. In two cases (patients 17 and 18 in Table II) there was a short-lived partial response with one of them showing tumour progression which had not quite achieved the UICC definition of PD at the time of completing the study (patient 18). The case was therefore recorded into a NC category. Patient 21 showed a change in the DI from 1.1 to 1.7 and an increase in SPF; tamoxifen treatment was ineffective in this patient.

In one case (patient 10) a sample taken 1 month after commencing tamoxifen showing a reduction in ploidy from aneuploid to diploid, with the latter sample being cellular and containing predominantly malignant cells. In this particular case a repeat FNA taken before therapy had also shown a diploid peak, but with a CV >10%, which was therefore removed from the analysis. This case clearly demonstrates the problem of intratumoral heterogeneity and no interpretation could be drawn on the changes in DNA content after tamoxifen therapy in the particular example.

### Discussion

*Reproducibility of FNAs*

When FNAs were taken at weekly intervals with no intervening therapy, there was good concordance in the DIs and SPFs measured on the two samples. However, it should be noted that no data are available on the variation of DI or SPF over periods longer than 1 week with no intervening therapy as this was not considered ethical in a clinical study. These data agree with several other reports (Prey *et al.*, 1985; Erhardt & Auer, 1986; Remvikos *et al.*, 1991) which have all shown good correlation between sequential FNAs from the same tumour. Only one study (Mullen & Miller, 1989) has commented on significant variation in DNA analysis caused by intratumoral heterogeneity between two fine-needle aspirates taken from the same tumour. Even here a comparison of DI between two needle aspirates showed less than 5% variation in 10 out of 11 cases.

Greater variation has been obtained when comparing FNAs with paraffin section from the same tumour (Greenbaum *et al.*, 1984; Prey *et al.*, 1985; Kalloniemi, 1988). This may explain why some studies in which both techniques have been employed have failed to obtain any consistent changes in DNA content in breast tumours after tamoxifen therapy (Baildam *et al.*, 1987).

These reported differences may also relate to other factors such as tumour size. It is more difficult to sample uniformly across a large tumour which is also more likely to be heterogeneous in terms of the proliferation characteristics of its individual cells. It should therefore be noted that in this study 18/21 of cases had tumours <5 cm in diameter. Also, considerable care was taken to sample widely from several areas within the same tumour. This is not possible when a paraffin-embedded tissue section is taken from the tumour after surgery if only one 40-μm section of tumour is taken for analysis.

The results of this study have shown that, providing any sample with a CV > 10% on flow cytometry or diploid with <100 malignant cells per cytospin slide was removed from the analysis, significant intratumoral heterogeneity was seen in only one case (patient 10).

In this pilot study, we were able to monitor changes in only 21 out of 27 patients. However, this was part of a multifactorial study. Material for 12 cytospins was taken from the aspirate before storing the remainder of the sample for flow cytometry. We would expect a higher success rate from aspirates taken for one cytospin (conventional cytological stain) and flow cytometry only. If these measurements are shown to be of real benefit in patient management, patient recall to obtain a repeat sample would be justified.

### *The use of an FNA to monitor treatment*

Considering the data from the FNAs taken sequentially during treatment, the clearest indication of successful therapy was a failure to observe malignant cells by conventional cytology in the post-treatment sample. This was also reflected in the flow cytometry in that only diploid cells were detected in the post-treatment samples of what had previously been recorded as aneuploid tumours (patients 1–3). However, in six patients, a reduction in the SPF was recorded by flow cytometry before the disappearance of malignant cells from the aspirate (patients 4–9).

In seven patients, tumours were shown to be aneuploid and sequential FNAs showed either no change or a rise in SPF. These cases showed eventual tumour progression with an increase in tumour size which in only one patient was still insufficient (<25%) to classify as PD. There was a median lead time of 7 months between the repeat FNA result and eventual tumour progression (see Table II, patients 15–21).

The difference in SPF between pre- and post-treatment samples for tumours achieving a complete response versus those only reaching a partial response or less was significantly different ($P<0.01$) even with the small numbers in the study.

In four patients (patients 11–14) a change from diploid to aneuploid was observed while the tumours were still in partial remission and on average after only 6 months of therapy. All these cases eventually showed clinical evidence of tumour progression. However, the change in ploidy was picked up with a mean lead time of 5 months before progression was detected clinically. This change was presumably caused by regression of the main diploid component and subsequent growth of a small aneuploid component already present within the tumour or from clonal development of a new cell population (Nowell, 1976). This is consistent with data (Kute *et al.*, 1985) showing that diploid tumours are more likely to be oestrogen receptor positive and hence respond to tamoxifen.

A change from diploid to aneuploid or a rise in SPF may reveal a failure in therapy even before changes in tumour size become evident. Conversely, a rapid decrease in the number of malignant cells in an FNA which was initially cellular or a fall in SPF may indicate a complete response. These data suggest that monitoring the changes in DI and SPF in patients on tamoxifen may enable the physician to select which patients are most likely to obtain a complete response or not to primary tamoxifen therapy. A larger study to investigate these changes further is therefore warranted.

We thank the Breast Cancer Research Fund and the Cancer Research Campaign for supporting this work.

### References

ALLEGRA, J.C., LIPPMAN, M.E., THOMPSON, E.B., SIMON, R., BARLOCK, A., GREEN, L., HUFF, K.K., DO, H.M., AITKEN, S.C. & WARREN, R. (1980). Estrogen receptor status: an important variable in predicting response to endocrine therapy in advanced breast cancer. *Eur. J. Cancer*, **16**, 323–331.

BAILDAM, A.D., ZALOUDIK, J., HOWELL, A., BARNES, D.M., MOORE, M. & SELLWOOD, R.A. (1987). Effect of tamoxifen upon cell DNA analysis by flow cytometry in primary carcinoma of the breast. *Br. J. Cancer*, **55**, 561–566.

BATES, T., RILEY, D., HOUGHTON, J., FALLOWFIELD, L. & BAUM, M. (1992). Tamoxifen alone is not adequate treatment for most elderly patients with operable breast cancer. *Breast*, **1**, 117–118.

BRUNNER, N., BRONZERT, D., VINDELOV, L.L., RYGAARD, K., SPANG-THOMSON, M. & LIPMANN, M. (1989). Effect on growth and cell cycle kinetics of oestradiol and tamoxifen on MCF-7 human breast cancer cells grown in in-vitro and in nude mice. *Cancer Res.*, **49**, 1515–1520.

CAMPBELL, F.C., BLAMEY, R., ELSTON, C.W., MORRIS, A.W., NICHOLSON, R.I., GRIFFITHS, K. & HAYBITTLE, J.L. (1981). Quantitative oestradiol receptor values in primary breast cancer and response of metastases to endocrine therapy. *Lancet*, **ii**, 1317–1319.

CLARKE, R.B., LAIDLAW, I.J., JONES, L.J., HOWELL, A. & ANDERSON, E. (1993). Effect of tamoxifen on Ki67 labelling index in human breast tumours and its relationship to oestrogen and progesterone receptor status. *Br. J. Cancer*, **67**, 606–611.

ERHARDT, K. & AUER, G. (1986). Mammary carcinoma: DNA analysis in areas showing different histological features in the same tumour. *Acta Pathol. Microbiol. Immunol. Scand., Sect. A*, **94**, 21–28.

FISHER, B., REDMOND, C., BROWN, A., WICKERHAM, D.L., WOLMARK, N., ALLEGRA, J., ESCHER, G., LIPPMAN, M., SAVLOV, E. & WITLIFF, J. (1983). Influence of tumour oestrogen and progesterone levels on the response to tamoxifen and chemotherapy in primary breast cancer. *J. Clin. Oncol.*, **1**, 227–241.

GREENBAUM, E., KOSS, L.G., SHERMAN, A.B. & ELEQUIN, F. (1984). Comparison of needle aspiration and solid biopsy technique in the flow cytometric study of DNA distributions of surgically resected tumours. *Am. J. Clin. Pathol.*, **32**, 559–564.

HAYWARD, J.L., CARBONE, P.P., HEUSON, J.C., KUMAOKA, S., SEGALOFF, A. & RUBENS, R.D. (1977). Assessment of response to therapy in advanced breast cancer. *Cancer*, **39**, 1289–1293.

KALLONIEMI, O. (1988). Comparison of fresh and paraffin embedded tissue as starting material for DNA flow cytometry and evaluation of intratumoural heterogeneity. *Cytometry*, **9**, 164–169.

KUTE, T.E., MUSS, H.B., HOPKINS, M., MARSHALL, F., CASE, D. & KAMMINE, L. (1985). Relationship of flow cytometry results to clinical and steroid receptor status in human breast cancer. *Breast Cancer Res. Treat.*, **6**, 113–121.

LYKKESFELDT, A.E., LARSEN, J.K. & CHRISTENSEN, I.J. (1986). Cell cycle analysis of oestrogen stimulation and anti-oestrogen inhibition of growth of the human breast cancer cell line MCF-7. *Breast Cancer Res. Treat.*, **7** (Suppl.), 83–90.

MULLEN, P. & MILLER, W.R. (1989). Variations associated with the DNA analysis of multiple fine needle aspirates obtained from breast cancer patients. *Br. J. Cancer*, **59**, 688–699.

ORMEROD, M.G. (1990). Analysis of DNA: general methods. In *Flow Cytometry: a Practical Approach*. Ormerod, M.G. (ed.) pp. 69-86. IRL at Oxford University Press: Oxford.

OSBORNE, C.K., YOCHMOWITZ, M.G. & KNIGHT, W.A. (1980). The value of oestrogen and progesterone receptors in the treatment of breast cancer. *Cancer*, **46**, 2884–2888.

OSBORNE, C.K., BOLDT, D.H. & ESTRADA, P. (1984). Human breast cancer cell cycle synchronization by oestrogens and anti-oestrogens in culture. *Cancer Res.*, **44**, 1433–1439.

NOWELL, P.C. (1976). The clonal evolutions of tumour cell populations. *Science*, **194**, 23–28.

PARIDAENS, R., SYLVESTER, R.J. & FERRAZZI, E. (1980). Clinical significance of the quantitative assessment of oestrogen receptors in advanced breast cancer. *Cancer*, **46**, 2889–2895.

PREY, M.U., MEYER, J.S. & STONE, K.R. (1985). Heterogeneity of breast carcinomas determined by flow cytometric analysis. *J. Surg. Oncol.*, **29**, 35–39.

REMVIKOS, Y., VIELH, P., PADOY, E., BENYAHIA, B., VOILEMOT, N. & MAGDELENAT, H. (1991). Breast cancer proliferation measured on cytological samples: a study by flow cytometry of S-phase fractions and BrdU incorporation. *Br. J. Cancer*, **64**, 501–507.

RUBENS, R.D. & HAYWARD, J.L. (1980). Oestrogen receptors and response to endocrine therapy and cytotoxic chemotherapy in advanced breast cancer. *Cancer*, **46**, 2922–2924.

SUTHERLAND, R.L., GREEN, M.D., HALL, R.E., REDDEL, R.R. & TAYLOR, I.W. (1983). Tamoxifen induces accumulation of MCF-7 human mammary carcinoma cells in the G0/G1 phase of the cell cycle. *Eur. J. Cancer Clin. Oncol.*, **9**, 615–621.

YOUNG, P.C.M., EHRLICH, C.E. & EINHORN, L.H. (1980). Relationship between steroid receptors and response to endocrine and cytotoxic chemotherapy in metastatic breast cancer. *Cancer*, **46**, 2961–2963.